# MiR-210 improves postmenopausal osteoporosis in ovariectomized rats through activating VEGF/Notch signaling pathway

**DOI:** 10.1186/s12891-023-06473-z

**Published:** 2023-05-18

**Authors:** Li-Jue Ren, Xiao-Hui Zhu, Jiu-Ting Tan, Xiang-Yu Lv, Yan Liu

**Affiliations:** 1 Department of Endocrinology, The First Affiliated Hospital of Baotou Medical College, Inner Mongolia University of Science and Technology, Baotou, Inner Mongolia China; 2 Soochow University, Gusu District, Suzhou City, Jiangsu Province China

**Keywords:** MiR-210, VEGF/Notch1 signaling pathway, Osteoporosis, Ovariectomized (OVX) rats

## Abstract

**Background:**

To explore the effect and mechanism of action of miR-210 on postmenopausal osteoporosis (PMPO) in ovariectomized rats in vivo.

**Methods:**

An ovariectomized (OVX) rat model was established by ovariectomy. Tail vein injection was performed to overexpress and knock down miR-210 in OVX rats, followed by the collection of blood and femoral tissues from each group of rats. And quantitative real-time polymerase chain reaction (qRT-PCR) was applied to assess the expression level of miR-210 in femoral tissues of each group. Micro computed tomography (Micro CT) was adopted to scan the microstructure of the femoral trabecula in each group to obtain relevant data like bone mineral density (BMD), bone mineral content (BMC), trabecular bone volume fraction (BV/TV), trabecular thickness (Tb.Th), bone surface-to-volume ratio (BS/BV), and trabecular separation (Tb.Sp). ELISA was used for determining the level of bone alkaline phosphatase (BALP), amino-terminal propeptide of type I procollagen (PINP), osteocalcin (OCN), and C-terminal telopeptide of type I collagen (CTX-1) in serum; and Western blot for the protein level of Runt-related transcription factor 2 (Runx2), osteopontin (OPN), and collagen type I alpha 1 (COL1A1) in femoral tissues.

**Results:**

MiR-210 expression was significantly decreased in femoral tissues of OVX rats. Overexpression of miR-210 could obviously increase BMD, BMC, BV/TV and Tb.Th, whereas significantly decrease BS/BV and Tb.Sp in femurs of OVX rats. Moreover, miR-210 also downregulated BALP and CTX-1 level, upregulated PINP and OCN level in the serum of OVX rats promoted the expression of osteogenesis-related markers (Runx2, OPN and COL1A1) in the femur of OVX rats. Additionally, further pathway analysis revealed that high expression of miR-210 activated the vascular endothelial growth factor (VEGF)/Notch1 signaling pathway in the femur of OVX rats.

**Conclusion:**

High expression of miR-210 may improve the micromorphology of bone tissue and modulate bone formation and resorption in OVX rats by activating the VEGF/Notch1 signaling pathway, thereby alleviating osteoporosis. Consequently, miR-210 can serve as a biomarker for the diagnosis and treatment of osteoporosis in postmenopausal rats.

**Supplementary Information:**

The online version contains supplementary material available at 10.1186/s12891-023-06473-z.

## Background

In recent years, the incidence of age-related diseases has been increasing annually with the serious aging population. Osteoporosis, a systemic bone disease, is characterized by low bone mass, deteriorative bone microarchitecture, elevated bone fragility and increased risk of bone fracture [1]. Clinically, osteoporosis is classified into primary, idiopathic and secondary osteoporosis [2]. According to the Chinese Center for Disease Control and Prevention, the total number of patients with osteoporosis in China in 2015 exceeded 160 million, with a prevalence of 12.4%, with the most affected individuals being postmenopausal women [3]. Postmenopausal osteoporosis (PMPO) is the most common type of primary osteoporosis in clinical practice. Postmenopausal women are more likely to suffer from osteoporosis (nearly 80%) due to many factors, such as aging and decreased estrogen [4–6]. In addition, the incidence of PMPO reaches around 50%, and it is difficult to cure; Worse, patients with PMPO show a younger tendency. [7, 8]. PMPO, as a global public health problem, is not only a serious mental and economic burden on society, families and patients, but also a challenge in the treatment of clinical departments such as orthopedics and endocrinology. Therefore, it is of great social significance and economic value to explore the pathologic mechanisms and molecular biomarkers for the diagnosis and treatment of menopausal osteoporosis.

MicroRNAs (miRNAs), a class of endogenous non-coding RNAs with 18–25 nucleotides in length, regulate gene expression by specifically binding to the 3’-UTR of target genes [9]. Moreover, miRNAs are involved in regulating a range of biological processes including cell differentiation, proliferation, invasion, metastasis, and apoptosis [10, 11]. Several studies have shown that abnormal expression of miRNAs can cause abnormal bone metabolism and then lead to osteoporosis. Yin et al. pointed out that miR-151a-3p was upregulated in osteoporosis, and it reduced femoral BMD and biomechanical parameters in OVX rats by targeting SOCS5 and activating JAK2/ STAT3 signaling, and then promoted PMPO [12]. Li et al. discovered that miR-23b-3p was upregulated in bone tissue of PMOP rats and promoted PMPO by targeting MRC2 and regulating the Wnt/β-catenin signaling pathway [13]. Additionally, several studies have revealed the role of some other miRNAs in alleviating osteoporosis. For example, Zhang et al. claimed that miR-187 accelerated osteogenic differentiation of human multipotent stromal cells (hMSCs) by directly regulating BARX2, played a significant therapeutic effect [14]. Wang et al. found that miR-655-3p inhibited the progression of osteoporosis by targeting LSD1 and activating the BMP-2/Smad signaling pathway [15]. Jiang et al. revealed that miR-218-5p promotes osteoblast differentiation and inhibits osteoclast formation through the ROBO1/DKK-1 pathway [16]. Therefore, it is convinced that miRNAs may be therapeutic targets for osteoporosis.

MiR-210 is a crucial target gene of hypoxia-inducible factor, which is closely related to angiogenesis.[17]. Specifically, miR-210 expression gradually increases under hypoxia, normoxia and the induction of vascular endothelial growth factor and its overexpression in endothelial cells can stimulate the formation of capillary tube-like structures and cell migration under [18, 19]. Studies have reported that miR-210 may promote osteogenic differentiation of bone marrow mesenchymal stem cells (BMSCs) by regulating the expression of VEGF, and can promote the formation of blood vessels and regulate bone regeneration [20]. In addition, it was reported that the Notch1-mediated signaling pathway is associated with the lower proliferation and differentiation capacity of BMSCs in postmenopausal patients with osteoporosis, which may be one of the reasons for the reduced bone mass in postmenopausal patients with osteoporosis [21]. However, the function of miR-210 in osteoporosis has not been proved. Therefore, a typical ovariectomized (OVX) rat model was constructed in this study to discuss the role of miR-210 in OVX rats. All in all, the objective of this paper was to provide scientific evidence and basis for the use of miR-210 as a therapeutic target for osteoporosis in OVX rats.

## Materials and methods

### Model rat construction and grouping treatment

Thirty female SD rats (body weight: 180–220 g; age: 6 weeks) were included in this study, and then a postmenopausal osteoporotic rat model was established by ovariectomy [22]. Specifically, thirty rats were randomly divided into 5 groups with 6 rats in each group after adaptive feeding, including Sham group, OVX group, OVX + NC group, OVX + miR-210 mimic group and OVX + miR-210 inhibitor group. The rats in each group were fasted for 12 h and then anesthetized intraperitoneally with a concentration of 2% pentobarbital sodium injection. After the rats lost their corneal reflexes and consciousness, all four limbs of the rats in four OVX groups were fixed in a supine position. The linea alba, 3.5 cm from the vaginal orifice, was then used as an incision and a longitudinal incision of 1.5-2 cm was made using a scalpel. Continually, the subcutaneous tissue was separated with blunt dissection, followed by an incision of muscularis and peritoneum. Finally, the abdominal cavity was opened, the uterus was found, and the ovary was removed. Then the OVX rats were treated four weeks after the establishment of the OVX model as follows: OVX group, OVX rats were injected intravenously into the tail vein with an equal volume of normal saline (5 µL). OVX + NC group, OVX rats were injected intravenously into the tail vein with a negative vector (5 µL). OVX + miR-210 mimic group, OVX rats were injected intravenously into the tail vein with miR-210 overexpression vector (5 µL). OVX + miR-210 inhibitor group, OVX rats were injected intravenously into the tail vein with miR-210 inhibitor (5 µL). All injections were given weekly for a total of 8 weeks. Whereas in Sham group, the adipose tissue near ovaries was excised at an equal ovarian volume before suturing. To avoid infection, all rats received intramuscular injection of penicillin G for 3 consecutive days after surgery. Incidentally, rats were allowed to eat and drink freely and were routinely housed throughout the experiment. All rats were sacrificed at the end of the processing cycle, and the femur and serum from each group were collected for further index testing.

### Micro computed tomography (Micro CT)

Micro computed tomography (Micro CT) was utilized for scanning the microstructure of femoral trabeculae in rats of each group, and the system SkyScan1276 for imaging whole femurs at 10 μm isotropic voxel size. Specifically, samples fixed in 4% paraformaldehyde were tested, and the scanning conditions included an X-ray tube potential (85 kv), an X-ray intensity (200 ja), and an exposure time (400 ms) for image reconstruction. A 3-D image was obtained from a 2-D image by distance transformation of the grayscale image. Then 3D and 2D analyses were performed using a software analyzer. Bone microstructure analysis was conducted in the region of interest, and finally, bone mineral density (BMD), bone mineral content (BMC), trabecular bone volume fraction (BV/TV), trabecular thickness (Tb.Th), bone surface-to-volume ratio (BS/BV), and trabecular separation (Tb.Sp) were determined [23].

### qRT-PCR

Femurs from each group were collected. And total RNA from femurs was extracted using Trizol (Sigma, USA), followed by the detection of the concentration and purity of total RNA with Nanodrop software. Next, cDNA was synthesized on the basis of the instructions of the reverse transcription PCR kit (Takara, Japan). Then the treated cDNA was collected to synthesize miR-210 according to the real-time PCR kit (Takara, Japan). The primer sequences used were shown in Table 1, and the data was analyzed by the 2^−ΔΔCt^ method.

**Table 1:** qRT-PCR primer sequences

Gene Name	Primer sequences (5’ to 3’)
miR-210	F: AGCGTGCTGTGCGTGTGAC
	R: CAGTGCAGGGTCCGAGGTATT
U6	F: GCTTCGGCAGCACATATACTAAAAT
	R: CGCTTCACGAATTTGCGTGTCAT

### Enzyme-linked immunosorbent assay (ELISA)

Blood from all rats was collected into coagulation-promoting tubes, then centrifugation at 1000 g was performed at 4 ℃ for 15 min. The serum was separated by a pipette for subsequent measurement. The level of bone alkaline phosphatase (BALP) kit, amino-terminal propeptide of type I procollagen (PINP), osteocalcin (OCN) and C-terminal telopeptide of type I collagen (CTX-1) in rat serum were assessed according to the instructions of Elisa kit (Nanjing Jiancheng, China).

### Western blot

The tissue proteins from each group of rat femurs were extracted on ice according to the instructions of RIPA lysis Solution (Solarbio, China). The protein concentration was determined by a BCA kit (Solarbio, China). The 30 µg protein was separated by Sodium Dodecyl Sulfate - Polyacrylamide Gel Electrophoresis (SDS-PAGE). After electrophoresis, the target protein was transferred to a polyvinylidene fluoride (PVDF) membrane, and the membrane was sealed in 5% skimmed milk powder for 2 h. Then diluted primary antibody (CST, USA) was added for incubation overnight in a shaker at 4 ℃. After washing three times with TBST, diluted secondary antibodies (ZSGB-BIO, China) were added for another 1-h incubation at room temperature. Finally, the protein bands were developed using enhanced chemiluminescence (ECL) chemiluminescence and then photographed and archived. Image-pro plus software was employed to calculate the grayscale of the bands, and the expression level of the target protein was analyzed with β-actin as an internal control.

### Statistics analysis

The data were expressed as mean ± standard deviation (SD). SPSS 24. 0 software was used for statistical analysis. The student t-test was used for comparing the differences between the two groups, and one-way ANOVA for comparing the differences between multiple groups. *P* < 0.05 was considered as the criterion for significant differences.

## Results

### Downregulation of miR-210 expression in femoral tissue of ovariectomized rats

**Fig. 1:**
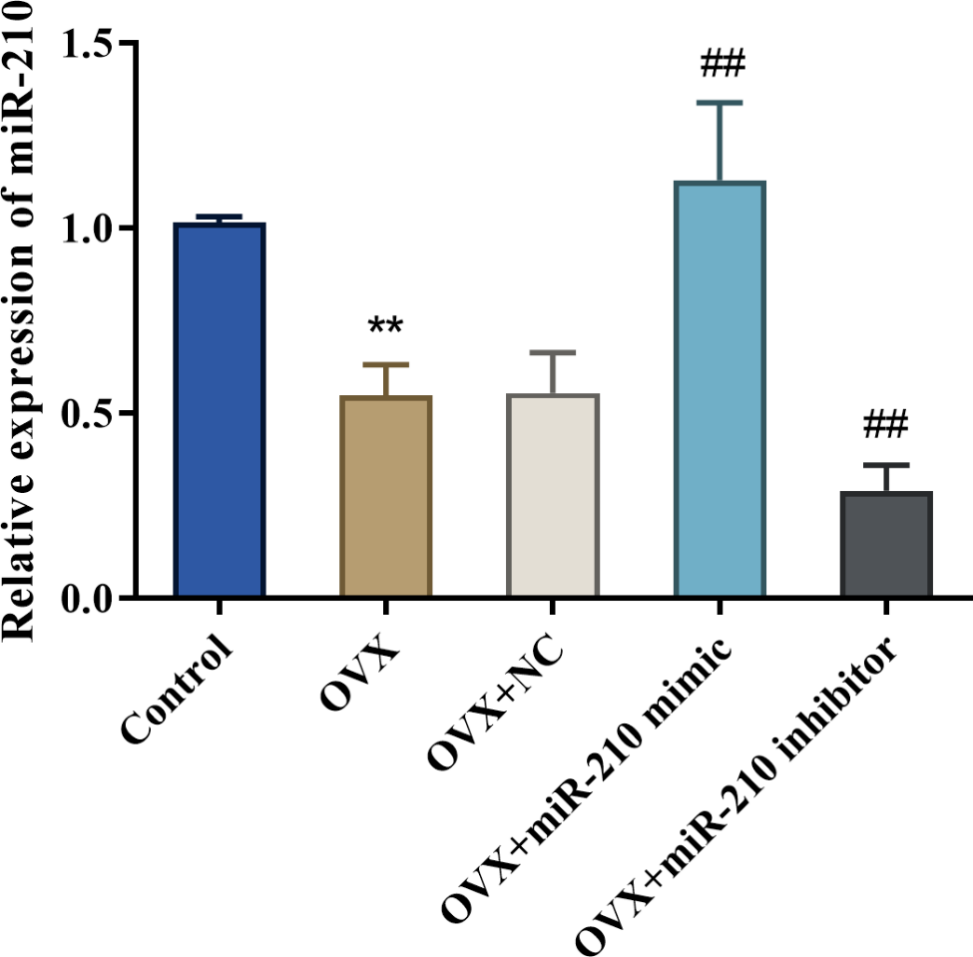
Downregulation of miR-210 expression in femoral tissue of ovariectomized rats. The expression level of miR-210 in the femoral tissue of rats in each group by qRT-PCR, **P < 0.01 vs. Sham group, ##*P* < 0.01 vs. OVX + NC group

In order to clarify the function of miR-210 in menopausal rats, we established a PMPO rat model (OVX group) by ovariectomy and overexpressed and knocked down miR-210 respectively by tail vein injection. The expression level of miR-210 in the femoral tissue of the OVX group was much lower than that of the Sham group (*P* < 0.01); in addition, tail vein injection of miR-210 mimic successfully elevated miR-210 expression level while tail vein injection of miR-210 inhibitor markedly inhibited miR-210 expression level in the femoral tissue of OVX rats (*P* < 0.01); and there was no significant difference in the expression of miR-210 between the OVX group and OVX + NC group (*P* > 0.05) (Fig. 1).

### Effect of miR-210 on the microstructure of femoral trabeculae in ovariectomized rats

**Fig. 2:**
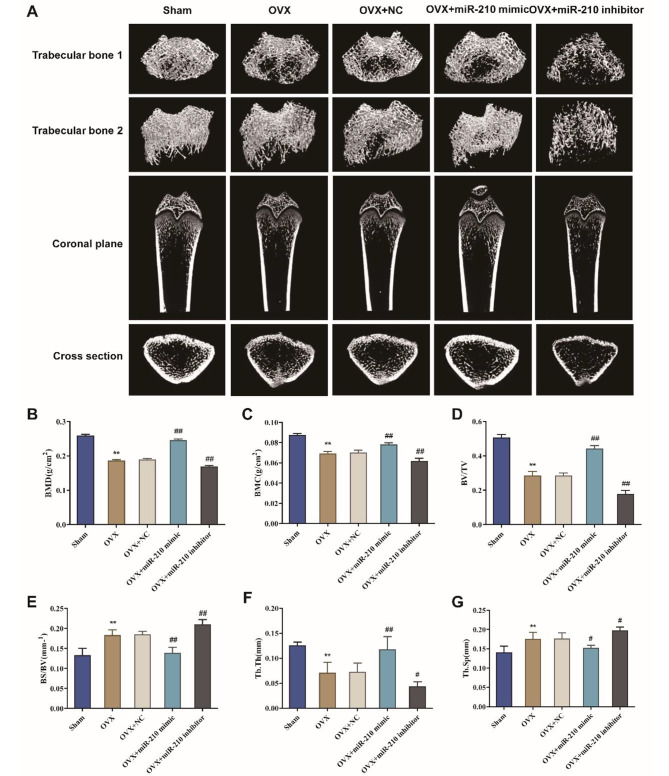
Effect of miR-210 on the microarchitecture of femoral trabeculae in ovariectomized rats. A: Representative Micro-CT images of 2D and 3D demonstrating that OVX-induced bone loss was improved by miR-210. B-G: Micro computed tomography (Micro CT) to measure bone mineral density (BMD, B), bone mineral content (BMC, C), trabecular bone volume fraction (BV/TV, D), bone surface-to-volume ratio (BS/BV, E), trabecular thickness (Tb.Th, F) and trabecular separation (Tb.Sp, G), **P < 0.01 vs. Sham group, #*P* < 0.05 and ##*P* < 0.01 vs. OVX + NC group

Micro-CT was used to detect the effects of treatment with miR-210 mimic or miR-210 inhibitor on bone mass loss in the OVX rats. Representative 2D and 3D Micro-CT images were shown in Fig. 2A. The results showed that the miR-210 improved the extensive bone mass loss in the OVX rat model. In addition, the effect of miR-210 on the femoral structure of OVX rats was assessed by measuring the indicators such as BMD, BMC, BV/TV, Tb.Th, BS/BV and Tb.Sp. The results revealed that, compared with the Sham group, BMD, BMC, BV/TV, and Tb.Th in the OVX group was significantly lower (*P* < 0.01), but BS/BV and Tb.Sp were obviously higher (*P* < 0.01). It indicated that a PMPO rat model was successfully constructed by ovariectomy. Besides, overexpression of miR-210 could significantly increase the BMD, BMC, BV/TV, and Tb.Th of the femur in OVX rats and notably decrease BS/BV and Tb.Sp; while knocking down of miR-210 obviously decreased BMD, BMC, BV/TV, Tb.Th and increased BS/BV and Tb.Sp (Fig. 2B-G). The above suggested that miR-210 could alleviate PMPO in OVX rats.

### Effect of miR-210 on the level of bone markers in the serum of ovariectomized rats

**Fig. 3:**
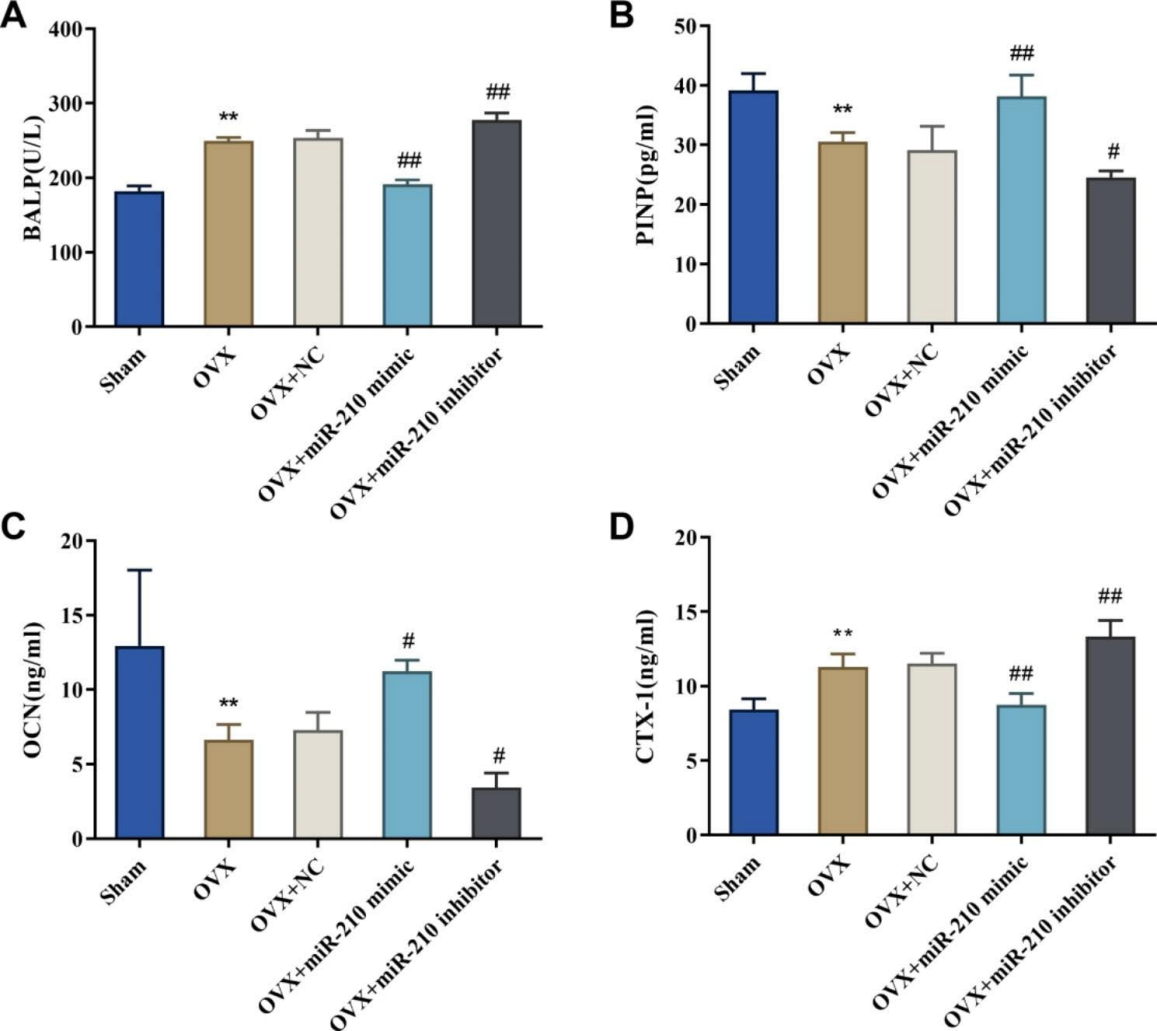
Effect of miR-210 on bone marker level in the serum of ovariectomized rats. A-D: The level of BALP (A), PINP (B), OCN (C) and CTX-1 (D) in serum of rats in each group were detected by ELISA, **P < 0.01 vs. Sham group, #*P* < 0.05 and ##*P* < 0.01 vs. OVX + NC group

Studies have indicated that bone turnover markers (BTM) such as BALP, PINP, OCN and CTX-1 are commonly applied for monitoring osteoporosis [24, 25]. Therefore, the levels of BALP, PINP, OCN, and CTX-1 in the serum of OVX rats were detected. And the outcomes showed that, in comparison with the Sham group, the level of BALP and CTX-1 was significantly higher (*P* < 0.01) while the level of PINP and OCN was obviously lower (*P* < 0.01) in the OVX group; the OVX + miR-210 mimic group exhibited significantly lower levels of BALP and CTX-1, while higher levels of PINP and OCN in serum compared with the OVX + NC group; and the level of BALP and CTX-1 in serum of OVX + miR-210 inhibitor group was significantly increased while the level of PINP and OCN was obviously decreased (Fig. 3A-D).

### Effect of miR-210 on the level of osteogenesis-related biomarkers in the femur of ovariectomized rats

**Fig. 4:**
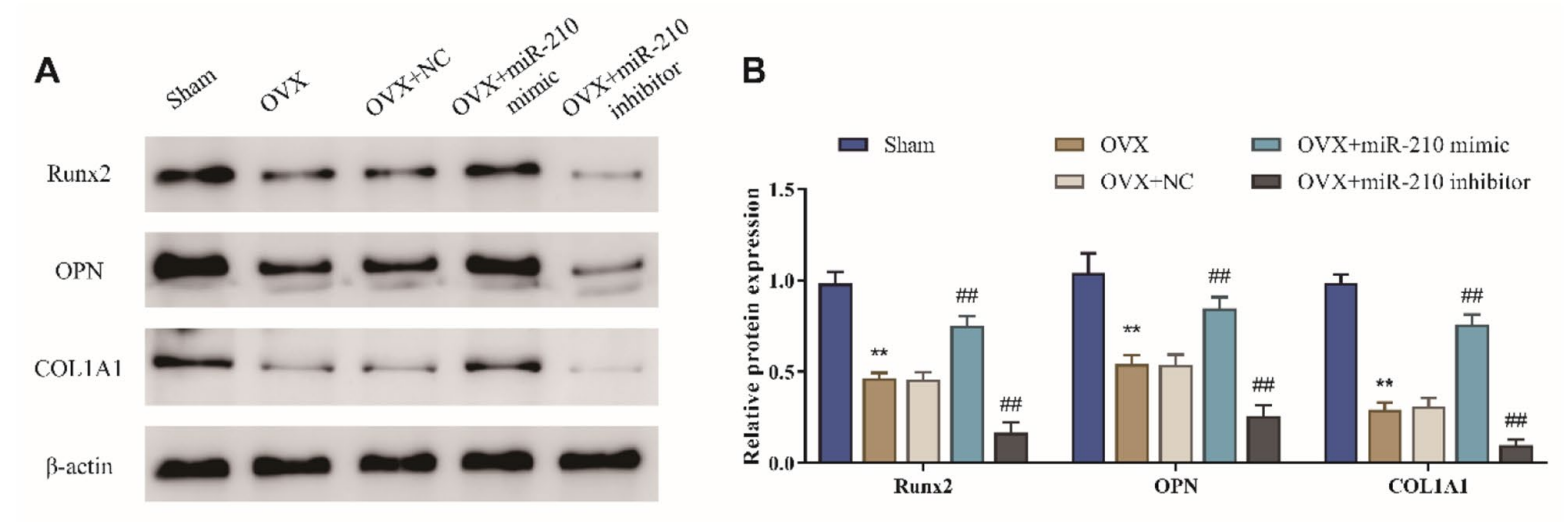
Effect of miR-210 on the level of osteogenesis-related biomarkers in the femur of OVX rats. A/B: The protein expression level of Runx2, OPN and COL1A1 in femoral tissues of rats in each group were detected by western blot, **P < 0.01 vs. Sham group, ##*P* < 0.01 vs. OVX + NC group

The effect of miR-210 on the expression of osteogenesis-related biomarkers such as Runt-related transcription factor 2 (Runx2), osteopontin (OPN), and collagen type I alpha 1 (COL1A1) was further assessed by Western blot. The protein level of Runx2, OPN and COL1A1 in the femur of OVX rats was obviously lower than that in the Sham group (*P* < 0.01); overexpression of miR-210 significantly increased the protein expression level of Runx2, OPN and COL1A1 in the femur of OVX rats, while miR-210 inhibitor markedly decreased the protein expression level of Runx2, OPN and COL1A1 (*P* < 0.01) (Fig. 4A/B). All of these indicated that miR-210 could significantly increase the expression of osteogenesis-related biomarkers in the femur of OVX rats.

### Effect of miR-210 on VEGF/Notch1 signaling pathway in the femur of OVX rats

**Fig. 5:**
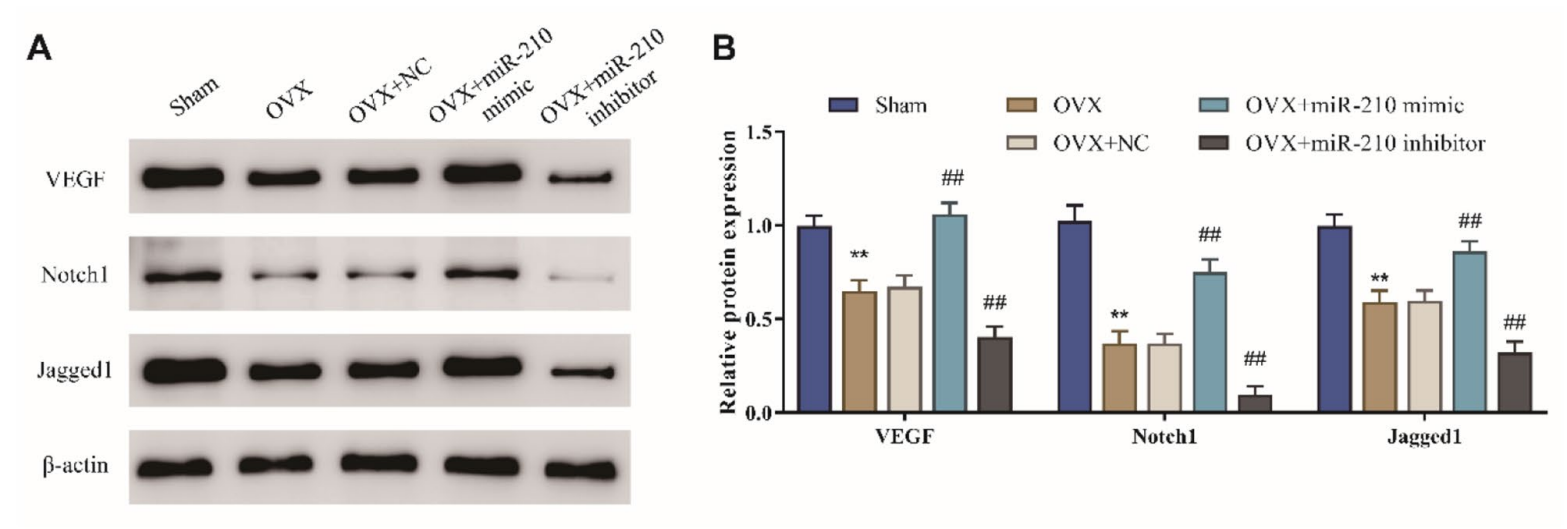
Effect of miR-210 on VEGF/Notch1 signaling pathway in the femur of OVX rats. A/B: The protein level of VEGF, Notch1 and Jagged1 in femoral tissues of rats in each group detected by western blot, **P < 0.01 vs. Sham group, ##*P* < 0.01 vs. OVX + NC group

Wang et al. stated that miR-210 induced protein expression of VEGF and Notch1 in HUVECs cells [26], and Lou et al. also proved that miR-210 overexpression could promote angiogenesis after cerebral ischemia by upregulating Notch1 signaling molecules [27]. To explore whether miR-210 also played a protective role in OVX rats by regulating the VEGF/Notch1 pathway, the expression of proteins associated with the VEGF/Notch1 pathway was examined. The results presented that the protein level of VEGF, Notch1 and Jagged1 in the femur of rats in the OVX group was much lower compared with that in the Sham group (*P* < 0.01). And the protein level of VEGF, Notch1 and Jagged1 in the femur of rats in the OVX + miR-210 mimic group was significant higher than that in OVX + NC group (*P* < 0.01), while that in the OVX + miR-210 inhibitor group was significantly lower (*P* < 0.01) (Fig. 5A/B). The above findings suggested that miR-210 could activate the VEGF/Notch1 signaling pathway in the femur of OVX rats.

## Discussion

Due to the increasing aging of the global population, the incidence of osteoporosis has increased substantially in recent years, and the mortality rate caused by osteoporotic fractures also reaches 15-30% [28]. Osteoporotic fractures significantly increase disability, morbidity and mortality [29]. In recent years, evidence has revealed the regulatory role of miRNAs in osteoblast growth, differentiation and function [30, 31]. BMD and BMC can effectively evaluate the degree of bone loss. Additionally, BV/TV and BS/BV can well reflect the bone mass of cancellous bone, bone cortex and the bone mass, respectively. As for Tb.Sp and Tb.Th, they are mainly adopted to assess the spatial morphological structure of bone trabeculae [32]. BALP, OCN and P1NP are markers of bone formation; CTXI is an indicator of bone resorption [24]. Runx2, OPN and COL1a1 serve as markers of osteogenesis [33]. Experimental animal models of osteoporosis are suitable tools for the study of osteoporosis, and among these models, the OVX rat model is the most commonly used [34]. In this study, a rat model of osteoporosis (OVX rat model) was also constructed by ovariectomy. OVX rats exhibited significant decreases in BMD, BMC, BV/TV, and Tb.Th, a notable increase in BS/BV and Tb.Sp in the femur, an obvious upregulation in the level of BALP and CTX-1, and a marked downregulation in the level of PINP, OCN, and the femur of Runx2, OPN, and COL1A1 protein level in the serum. These results are consistent with previous osteoporosis research [35]. The above outcomes of this study suggested that a PMPO rat model was successfully established by ovariectomy.

In this study, the expression of miR-210 was significantly downregulated in the femoral tissues of OVX rats, indicating that miR-210 may be involved in regulating osteoporosis in OVX rats. Overexpression of miR-210 significantly increased BMD, BMC, BV/TV and Tb.Th and decreased BS/BV and Tb.Sp in femurs of OVX rats; while after inhibition of miR-210, BMD, BMC, BV/TV and Tb.Th were decreased, and BS/BV and Tb.Sp were increased. Further, overexpression of miR-210 significantly decreased the level of BALP and CTX-1 in the serum of OVX rats, and significantly increased the level of PINP, OCN and the protein level of Runx2, OPN and COL1A1 in the femur, while after inhibition of miR-210, BALP and CTX-1 were increased, and PINP, OCN, Runx2, OPN and COL1A1 were decreased. These findings are consistent with previous results, such as Garmilla-Ezquerra et al. also found that the miR-210 expression in the discovery stage was close to statistical significance (*p* = 0.06) [35]. Meanwhile, Mizuno et al. revealed that miR-210 level was upregulated during osteoblastic differentiation, and it positively regulated osteoblastic differentiation of mouse mesenchymal ST2 cells [16]. The results above showed that miR-210 was associated with osteoblast activity in osteoporotic.

As is known to all, VEGF not only is one of the most vital molecules regulating vascular development and angiogenesis but also plays a key role in bone development [36]. Research has demonstrated that VEGF protein expression in tibial metaphysis is significantly reduced in patients with ovariectomy-induced osteoporosis [37]. The increased expression of VEGF in the periosteum and angiogenesis of osteoporotic rats may be closely related to the increased bone resorption activity in the periosteal regions [17]. Notch, as a pivotal regulator of vascular density, not only inhibits the proliferation of endothelial cells but also regulates the expression of VEGF receptors. The absence or ectopic activation of Notch signaling leads to an increase or decrease in vascular density, respectively [38]. Sun found that Notch could regulate the mineralization of osteoblasts by regulating the expression of Runx2, OPN and COL1A1 [39]. In addition, previous studies have shown that miR-210 plays a key role through VEGF/Notch signaling pathways in neuronal apoptosis in cerebral infarction [40] and angiogenesis in cerebral ischemia-reperfusion [41]. In this study, protein levels of VEGF, Notch1 and Jagged1 were significantly elevated in OVX rats with overexpression of miR-210, while after inhibition of miR-210, the above protein level was significantly decreased. It is suggested that overexpression of miR-210 may ameliorate osteoporosis in menopausal rats by activating the VEGF/ Notch1 signaling pathway.

In summary, we constructed an OVX rat model and found that miR-210 was decreased in OVX rats and its high expression could alleviate osteoporosis in postmenopausal rats. We have explored the role and mechanism of miR-210 in postmenopausal osteoporosis in vivo for the first time, but there are still the following limitations: the specific mechanism of miR-210 was obscure because only the VEGF/Notch1 signaling pathway activity was determined after knockdown or overexpression of miR-210, and it is not clear whether miR-210 also plays a role in improving osteoporosis through other signal pathways in this study. Meanwhile, we have not further verified this mechanism through pathway inhibitors. In addition, miRNAs mainly act on target genes. It’s unclear whether miR-210 can activate the VEGF/Notch signal pathway by targeting downstream target genes, thus alleviating osteoporosis. Further experiments are needed to explore and verify the above findings in order to provide a more detailed theoretical basis for miR-210 as a marker of osteoporosis.

## Conclusion

In conclusion, miR-210 expression is significantly downregulated in femoral tissues of OVX rats, and its high expression improves the microstructure of bone tissue, regulates bone formation and resorption in OVX rats, and then alleviates osteoporosis. Besides, mechanistic studies suggest that it may play these roles by activating the VEGF/ Notch1 signaling pathway. MiR-210 can serve as a diagnostic and therapeutic biomarker for the treatment of osteoporosis in postmenopausal rats.

## Electronic supplementary material

Below is the link to the electronic supplementary material.

Supplementary Material 1

## Data Availability

The dataset supporting the conclusions of this article is available at our institution contacting the corresponding author.

## Abbreviations

PMPO: postmenopausal osteoporosis
OVX: ovariectomized
Micro CT: Micro computed tomography
BMD: bone mineral density
BMC: bone mineral content
BV/TV: trabecular bone volume fraction
Tb.Th: trabecular thickness
BS/BV: bone surface-to-volume ratio
Tb.Sp: trabecular separation
BALP: bone alkaline phosphatase
OCN: osteocalcin
OPN: osteopontin
COL1A1: collagen type I alpha 1
VEGF: vascular endothelial growth factor
