# Supplementary material for: MiR-210 improves postmenopausal osteoporosis in ovariectomized rats through activating VEGF/Notch signaling pathway
Source: BMC Musculoskelet Disord. 2023 May 18;24:393. doi: 10.1186/s12891-023-06473-z (PMC10193724; doi:10.1186/s12891-023-06473-z)

Fugure 4A-Runx2

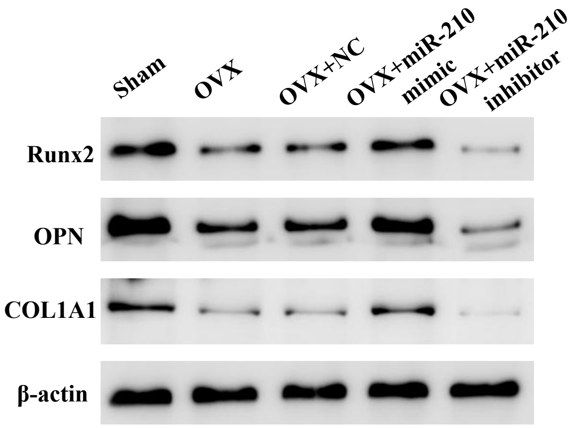

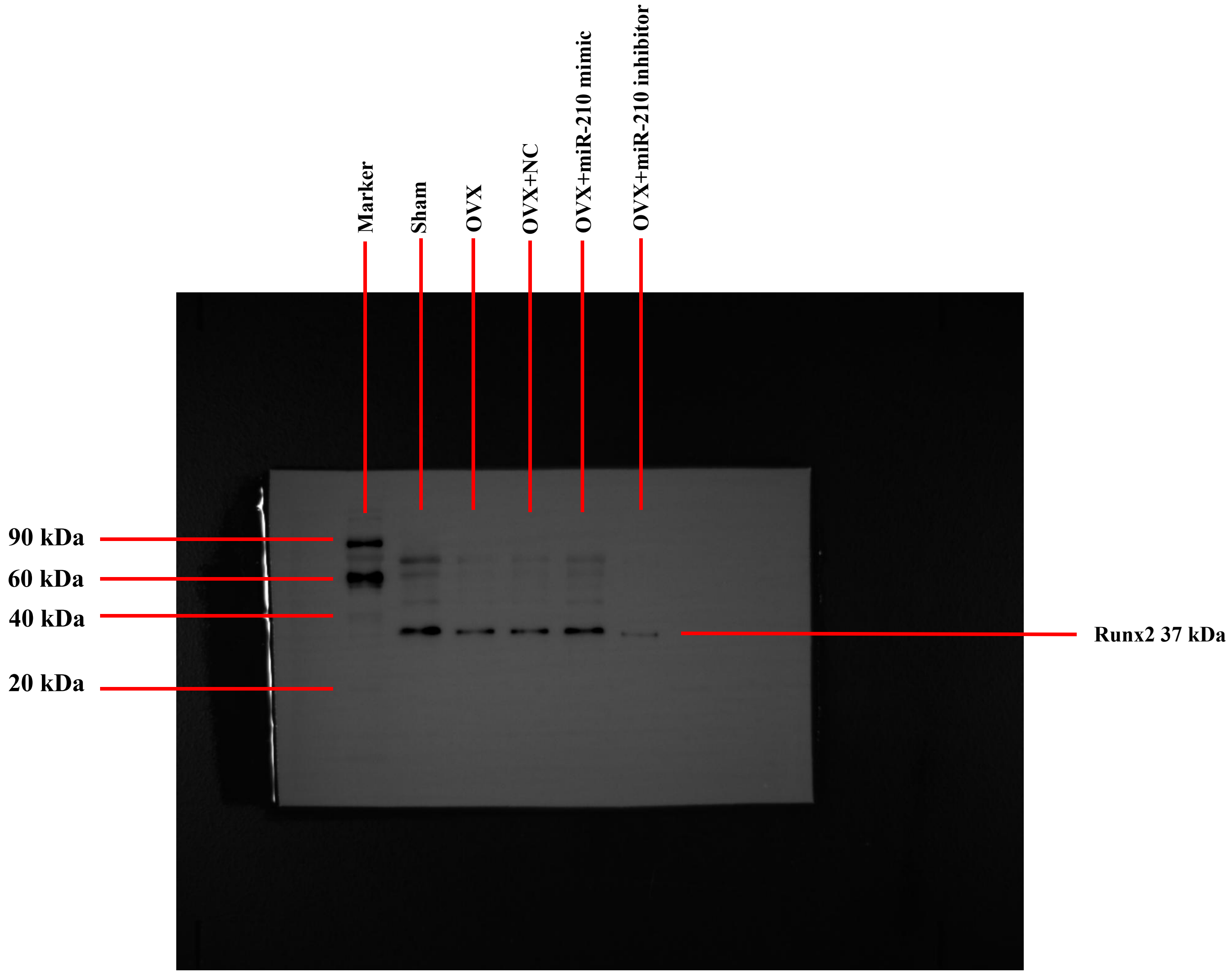

Fugure 4A-OPN

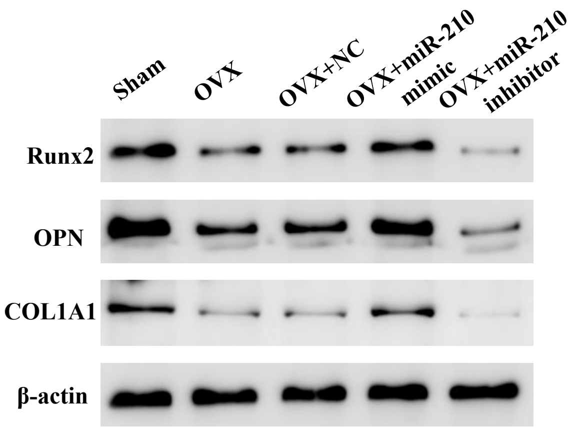

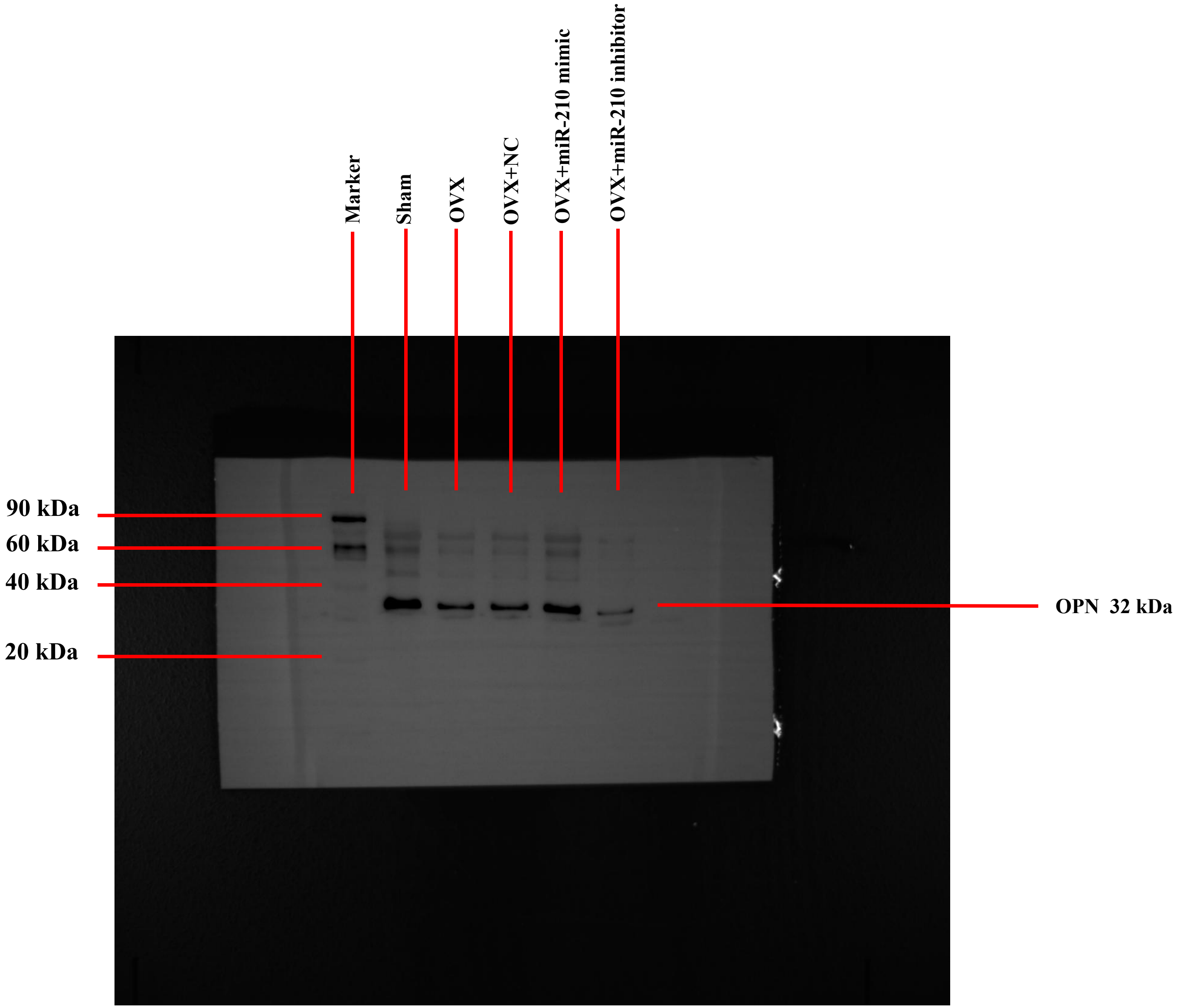

Fugure 4A-COL1A1

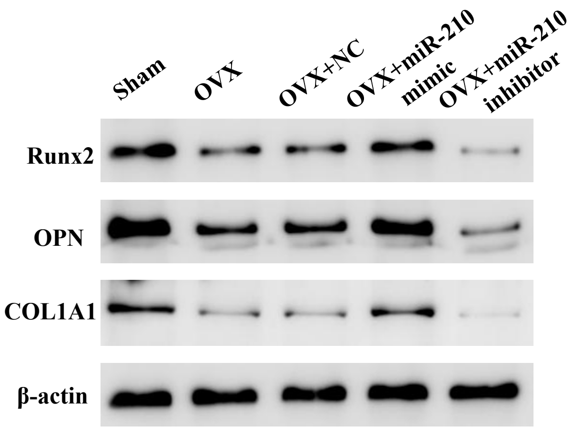

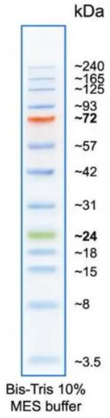

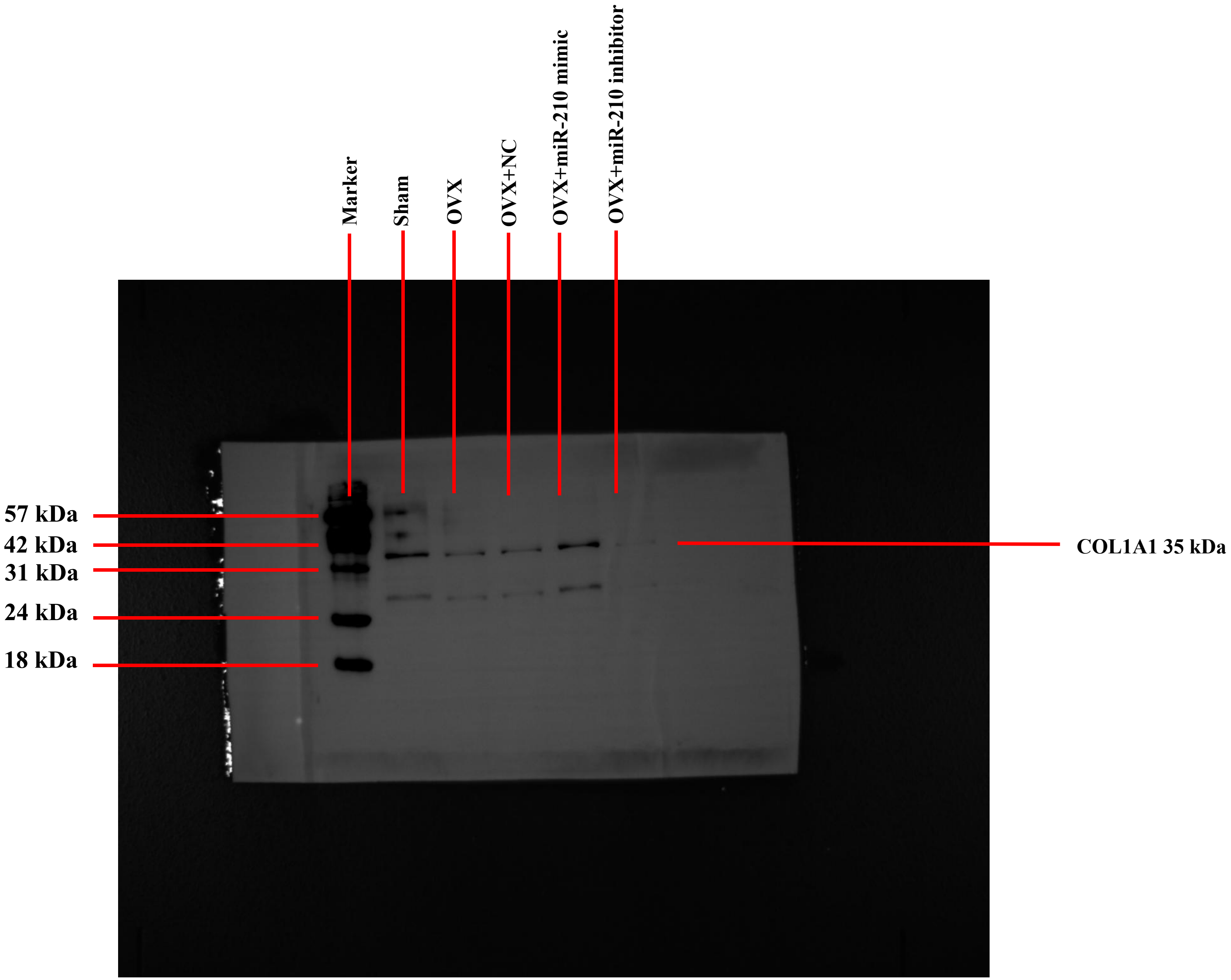

Fugure 4A-β-actin

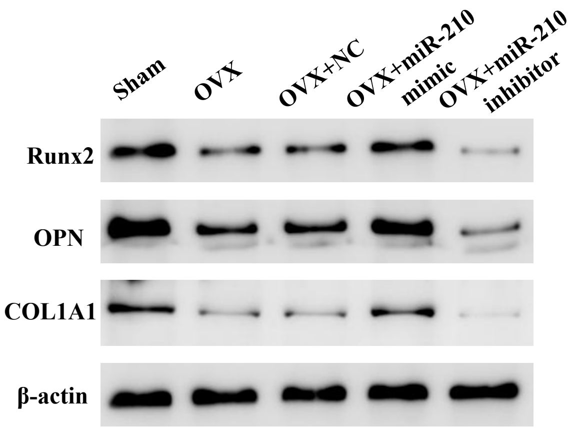

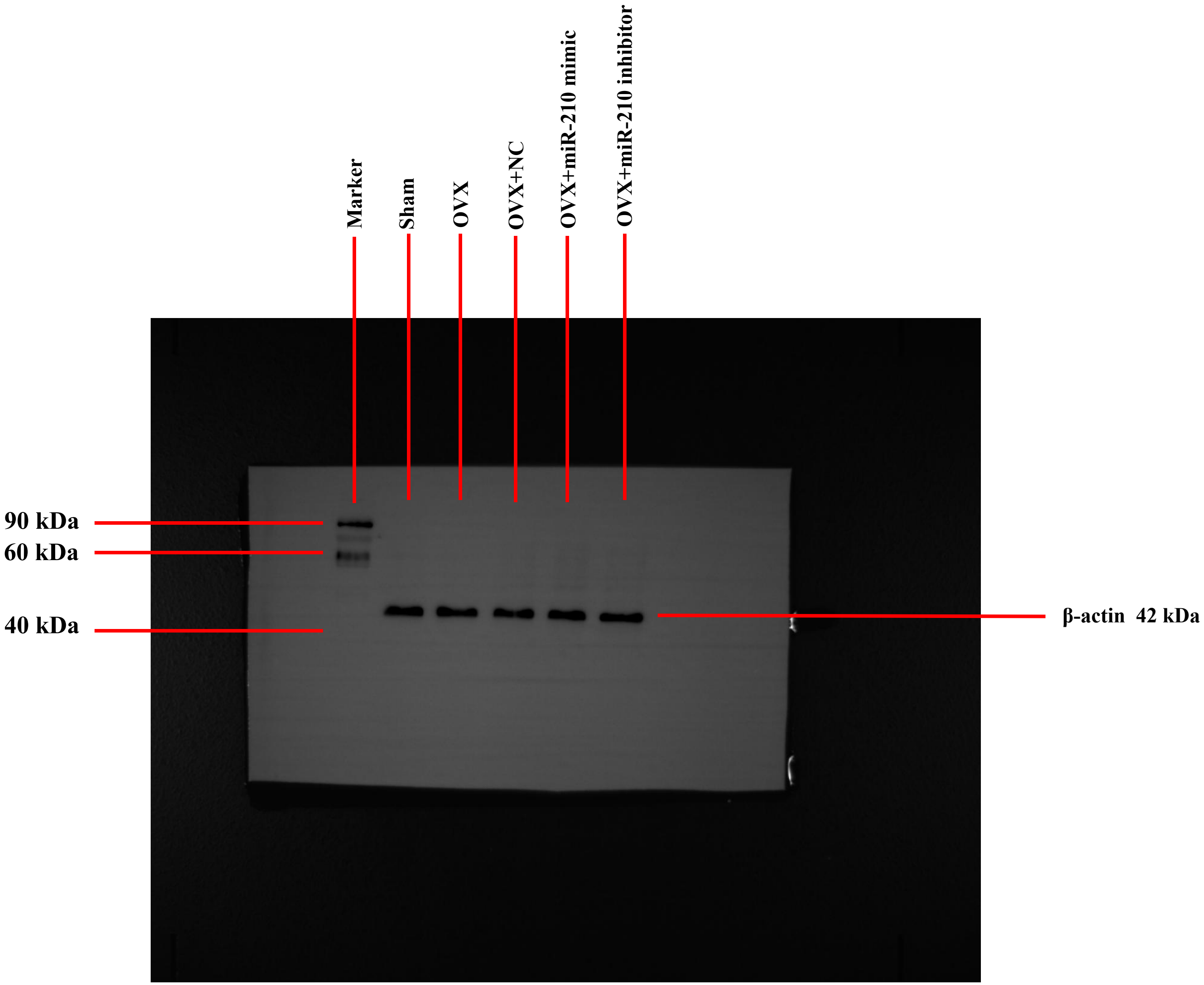

Figure 5A-VEGF

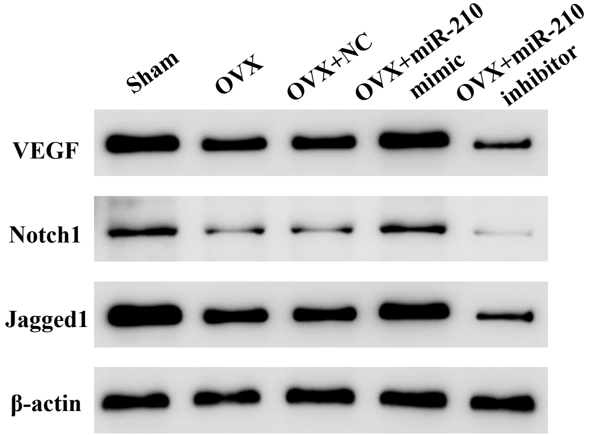

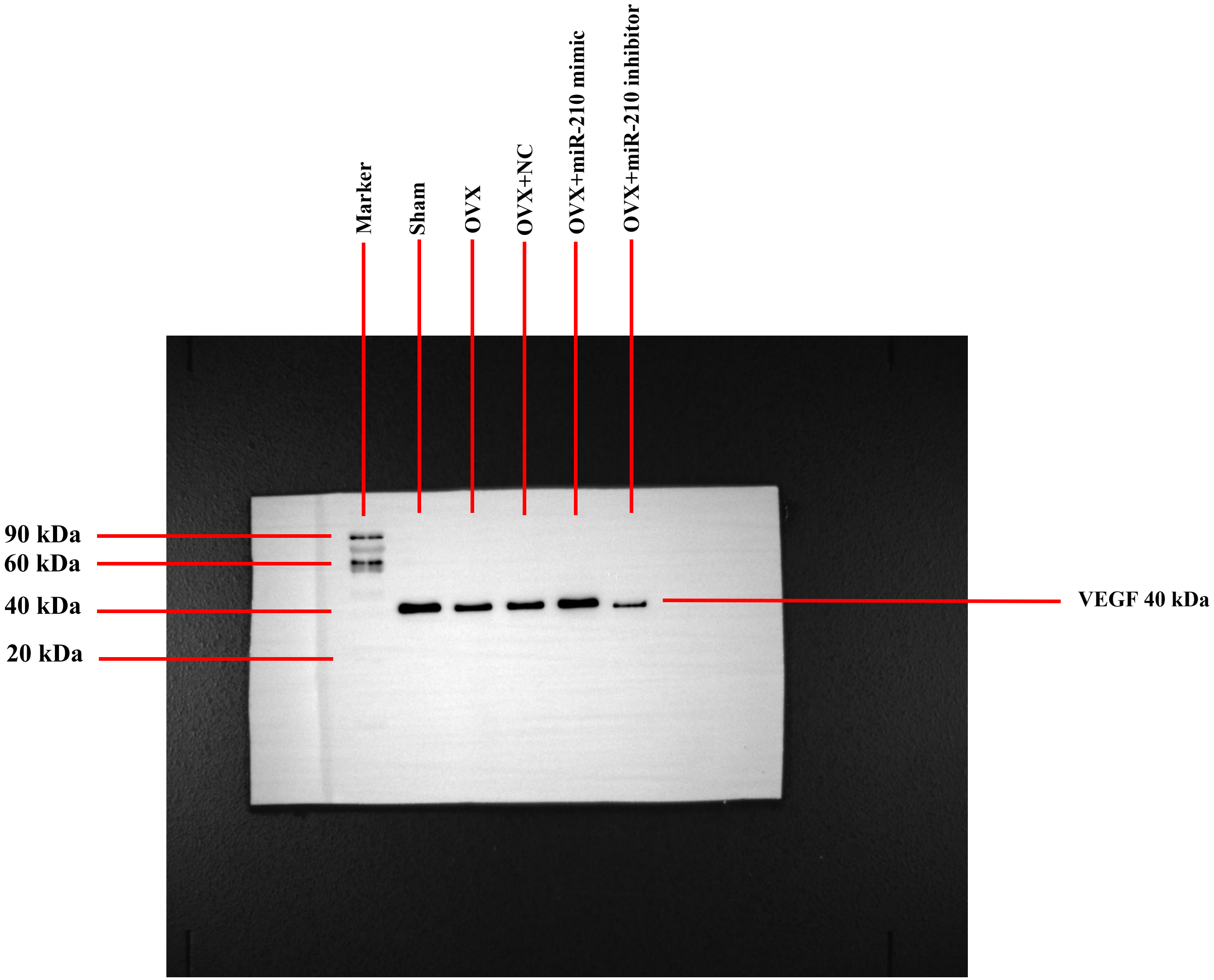

Figure 5A-Notch1

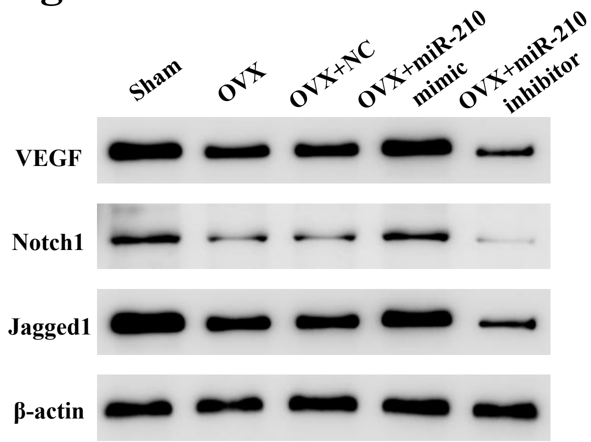

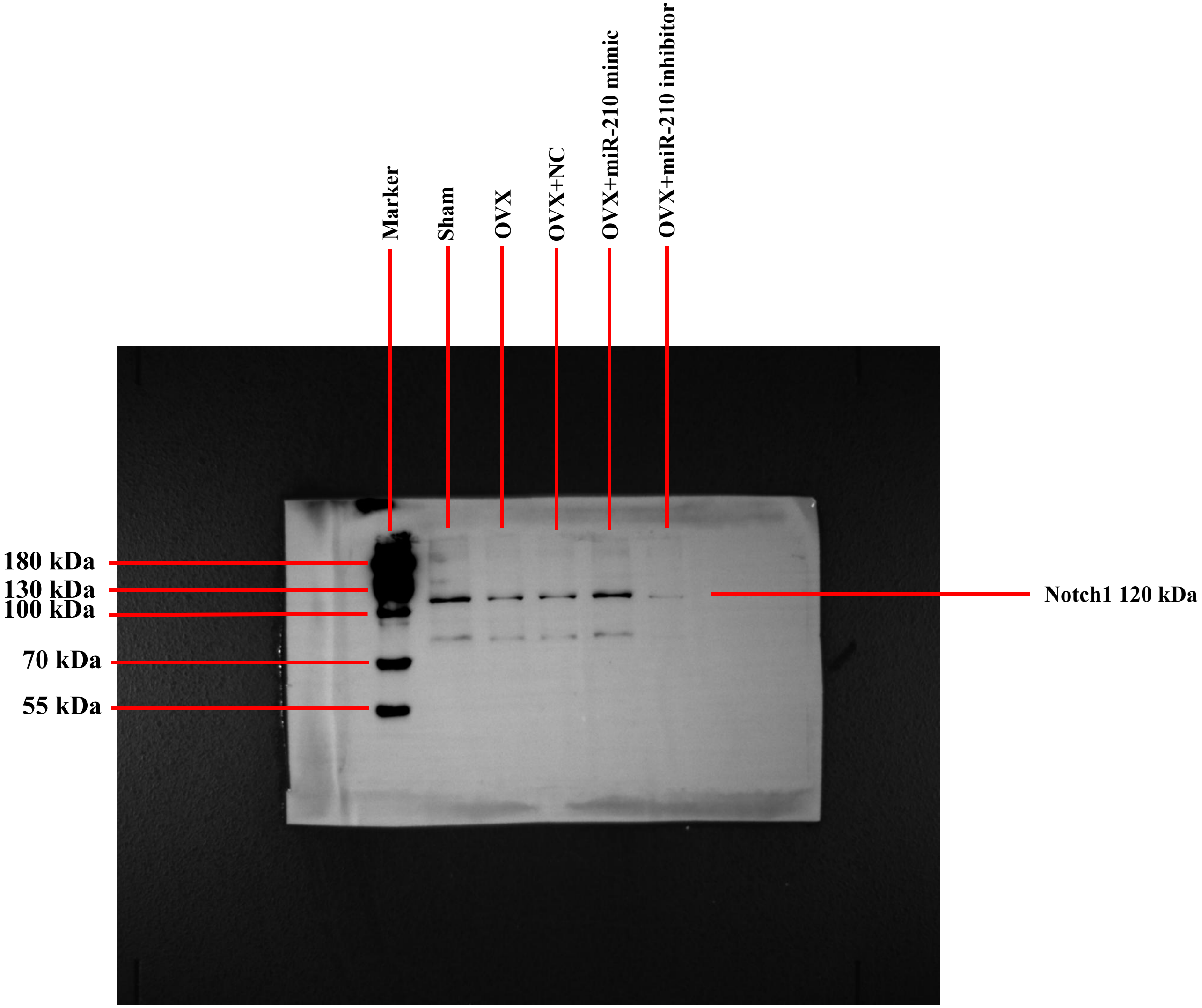

Figure 5A-Jagged1

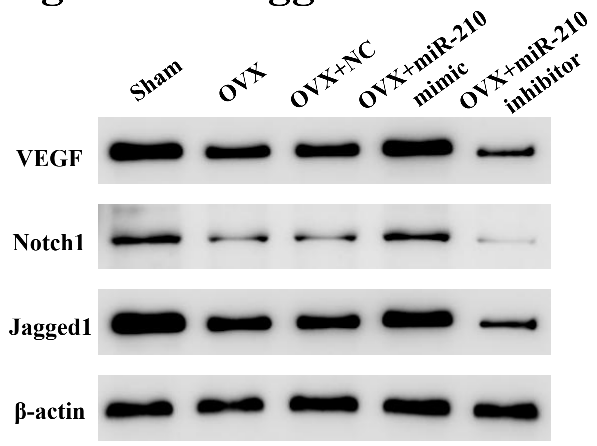

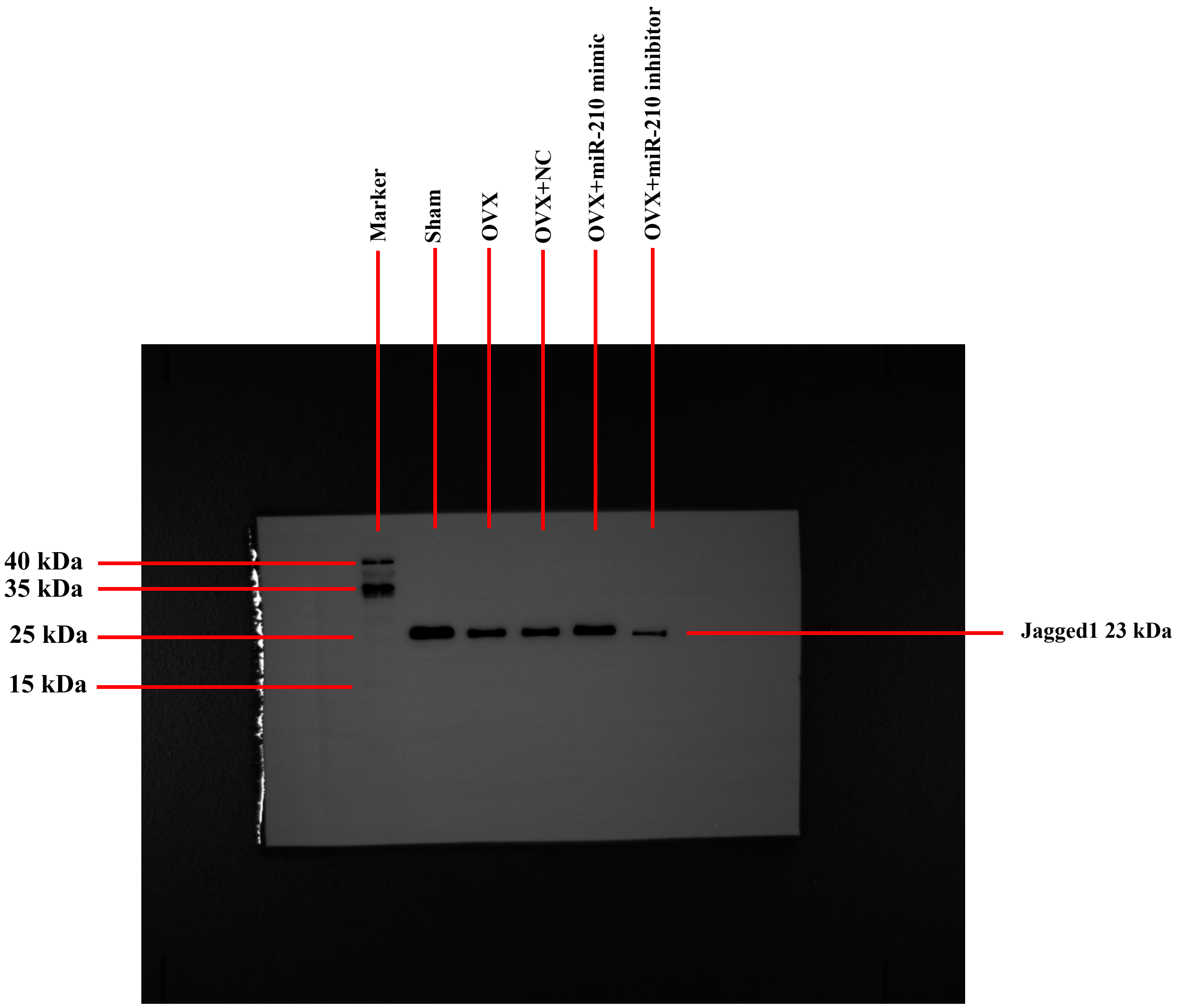

Figure 5A-β-actin

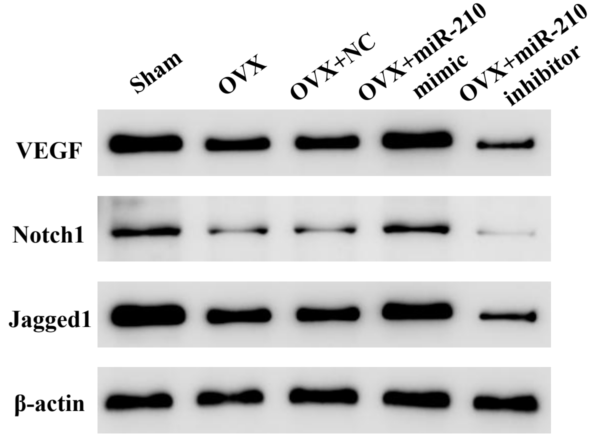

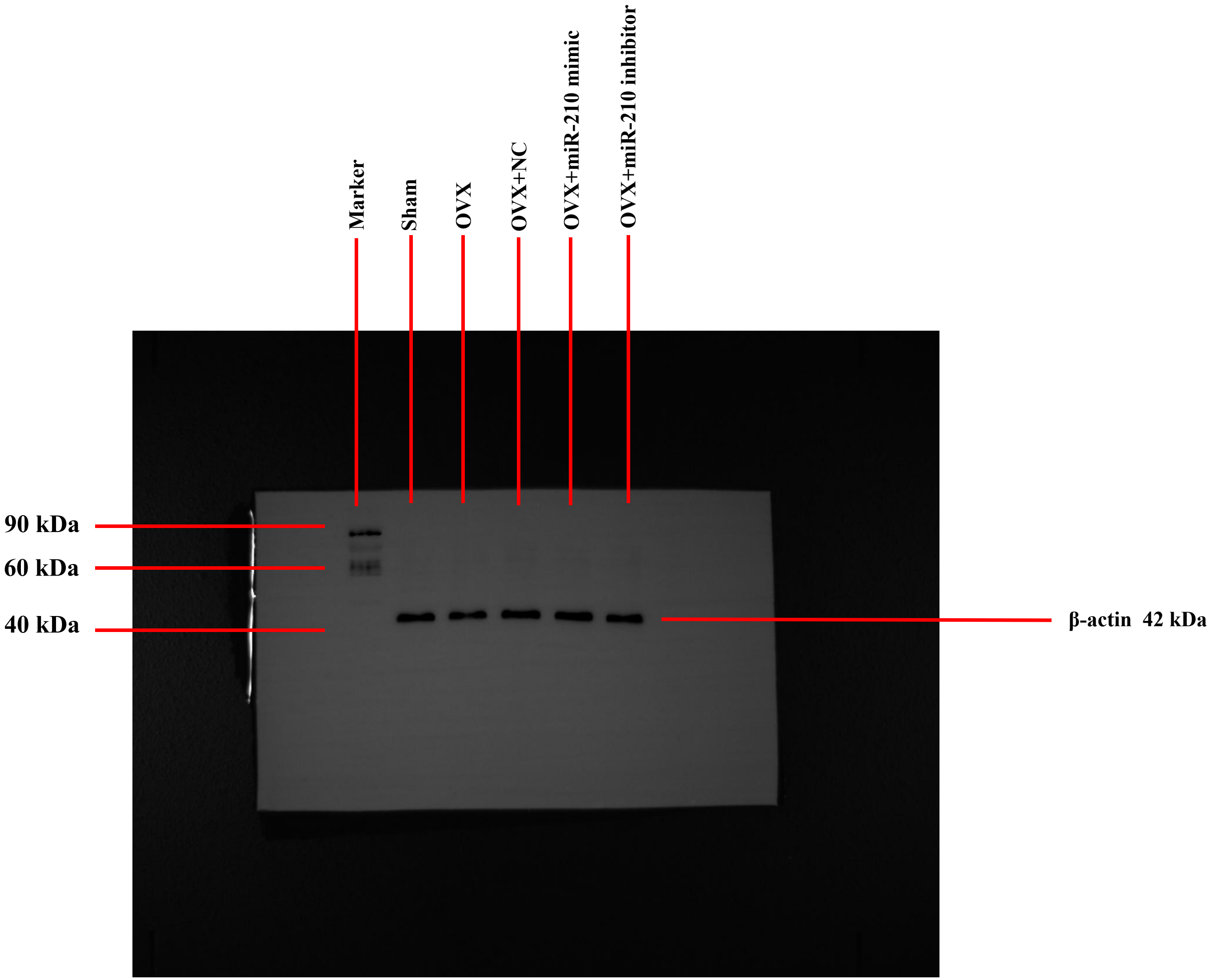

Supplement: Supplementary file 1 — Supplementary Material 1 [file 12891_2023_6473_MOESM1_ESM.pdf]
